# New York Odontological Society

**Published:** 1891-08

**Authors:** 

**Affiliations:** New York Odontological Society


### Reports of Society Meetings.

### NEW YORK ODONTOLOGICAL SOCIETY.

A REGULAR meeting of the New York Odontological Society was held on Tuesday evening, May 19, 1891, at the Academy of Medicine, No. 17 West Forty-third Street, New York City.

The President, Dr. William H. Dwinelle, in the chair.

*Dr. Perry.*—Mr. President, Professor Wight, who recently read a paper before us on the subject of "Work," presents to this Society a little book entitled "Suggestions to the Medical Witness."

*Dr. Allan.*—I move that we accept the book and tender to Professor Wight a vote of thanks.

Carried.

*The President.*—Since last we met, as you will perceive, gentlemen, a very interesting osteological collection, probably unparalleled in the world, has been placed in our rooms, collected through the energies and untiring industry, for many years, of our friend Dr. W. C. Barrett, of Buffalo, N. Y. This collection has been purchased of the scientific institution in Rochester, N. Y., where it has been owned, and has, I understand, been presented to the New York Odontological Society by its purchasers, who are, with few exceptions, members of the Society. The collection is to be free to dentists of the world for scientific research. We are all more or less aware of Dr. Barrett's services. He has been untiring in bringing together and focalizing this collection, which has been the contribution of science throughout the world. We have it now in our possession, and we hope and expect that it will be the nucleus for a collection whose superior has never been seen. Dr. Barrett, instead of going to Buffalo to-night, has stayed with us to arrange the specimens in their proper order in the cases provided. We would all like to hear a few words from Dr. Barrett.

*Dr. W. C. Barrett.*—I only wish that I could persuade myself that I am worthy the kind and complimentary terms which your honored president has used in alluding to me. I love this kind of work; I love this superb collection, and because of that I have spent the last three days in putting it in order. On my arrival I found the cases ready, or nearly so, and I have busied myself in arranging the specimens in them. The work is not yet completed, for they are but hurriedly crowded in, that they might be exhibited at this meeting.

And now may I say a few words concerning the arrangement? There are certain scientific demands to be met in the placing of such a systematic series as this. It is not a mere heterogeneous collection of objects, but it is a scientific illustration of the various orders and species. There are no duplicates here, but each one has its own definite place in the series. The great grizzly bear and the raccoon belong to the same class, and must be together, though they may to the uneducated eye look ill-assorted. The specimens are not to be put up to look pretty, but that each may best subserve the purposes for which it is intended, in connection with the rest. They are not to be classified according to size or general appearance, but each must follow a scientific and recognized law. The various orders must follow each other consecutively, and the species be properly arranged under them. The whole is intended for scientific study, and it must be arranged accordingly. This is somewhat interfered with by the fact that certain very large specimens cannot be contained in the cases. These fifteen-feet jaws of *Physeter Macrocephalus*, for instance, cannot be placed with the other Cetacea.

The series commences here (illustrating): On these shelves are the Primates, commencing with the species Homo, followed by the Simiadæ, Cercopithecidæ, Cebidæ, and Lemuridæ. All the Primates have a diphyodont-heterodont dentition. These are succeeded by the order Carnivora, divided into the Cynoidea, Eluroidea, Arctoidea, etc.

On these shelves are found the Ungulata, divided into the Perissodactyla and the Artiodactyla, and the Ruminants separated from the non-Ruminants.

In this case are the Rodentia in their proper order. Then follow the Marsupialia, the Cheiroptera, the Insectivora, the Cetacea, and Sirenia, and here are the sharks and rays, with the rest of Pisces. Here are the Reptilia and Batrachia, and here is a specimen in Aves,—a fossil bird that had unmistakable teeth.

This case is devoted to the fossils of the collection, and here may be found skulls of the cave bear, connected with the troglodyte period of man; the oreodon, an ungulate possessing peculiar characteristics; a giant rodent, Castoroides Ohioensis, and many others, together with a series of casts of long extinct mammals.

This case is made up entirely of prepared specimens; sections illustrating the manner of the insertion of different kinds of teeth, with the tissues of some arranged in a seemingly fantastic manner, pathological specimens, and rare forms of teeth. In this case are specimens of that curious substance, baleen, or whalebone, that anomalous formation which takes the place of the rudimentary teeth that are found alone in the fœtus of the right whale. It will be observed that some of the cases are crowded very much, while in others there is room to spare. This could not be avoided, as the cases were not specially constructed for the different orders, but were all made of the same size.

The specimens are not yet fully arranged, and there must still be considerable work expended upon the collection. There are no labels for the orders and species, and these must be made. But when it is finally finished, you need not fear to introduce any scientifically educated man, for he will, I hope, find it all arranged according to a definite plan. That method may be a little novel to some scientists, but it must be remembered that this is a dentists' museum, and the individual specimens are arranged to show the dentition.

I do not think that such a collection exists anywhere else, for none has been made, to my knowledge, to illustrate the dentitions of the different orders and classes. I am sure that it will be an inspiration to students, and it will be a credit to you of New York. Its placing here has been a labor of love with me, and my heart will always be in it. I hope to see it grow into greatly enlarged proportion. Dentists should study the scientific aspects of their own specialty, and I want to see the time when it will not be necessary to specify the species when one speaks of the crescentic molars of some of the Ungulata, for all will know exactly to what allusion has been made. All the spare time that I have had since this collection has been got together has been spent in it, and I am only sorry that I have to go so far to reach it.

I have the keys of the cases, which, when the work is completed, will be handed over to the proper authorities.

*The President.*—Gentlemen, you see Dr. Barrett fully justifies me in all I have said. I think this collection will be a great satisfaction to us all, and will be a means of instruction, and make New York a sort of Mecca to which scientific men will come and study the subjects that are introduced in this collection.

*Dr. Barrett.*—All work of preparation, placing the specimens on their proper stands, and mounting them, was done at Ward's Natural History Establishment, at Rochester, and the credit belongs to him.

*Dr. Phillips.*—I move a vote of thanks to Dr. Barrett for coming here and spending his time in arranging this collection.

The motion was carried.

### REPORT OF COMMITTEE ON THE DEATH OF DR. ATKINSON.

A man has passed away, one who filled a large place in our profession, and it is fitting that this society should take notice of the event in a proper manner, not in the way of an obituary notice or eulogy, but to place on record a brief statement of our reasons for mourning his loss and reviving his memory.

In life no one was more devoted to his profession than our lost friend. In fact, it was all in all to him, and he threw his very being into it. The best was nearly good enough for him,—never too good. He labored in season and out of season for the advancement of his chosen field of labor, and never knew what it was to spare himself. What he believed to be right he wanted all others to see as he did, and so he was ever a teacher, and always taught that which was best and most true to *nature*, and his instruction was free to all.

As a man he was tender and generous to a fault. Easily imposed upon, but never bearing malice, his house and purse were open to all.

Therefore it is we think of him kindly, lovingly, and hope that the angels he so fully believed in and prayed to when he was with us now minister to him and have lifted the burden from his weary soul, and opened his eyes in a world of bliss.

GEO. S. ALLAN.
BENJAMIN LORD.
A. L. NORTHROP.

*Dr. Jarvie.*—I move that the report be accepted and entered in full upon a memorial page which shall be set apart for the purpose in our minute-book.

Seconded and carried.

*Dr. Perry.*—Another great light in our profession has recently gone out. I refer to the recent death of Dr. Edward Maynard. It seems to me that the Society should not allow his death to pass without some recognition of his great services to our profession, and I move that a committee be appointed by the chair to draft suitable resolutions.

Seconded and carried.

*The President.*—I appoint, as that committee, Drs. Perry, Francis, and Lord.

*Dr. Perry.*—I think it eminently fitting that our president be added to that committee, and, gentlemen, I put it to the house, if he should be added to that committee; and also that he be made its chairman. He and Dr. Maynard were boys together.

Motion carried.

*Dr. Perry.*—A request came to us from the New Jersey Society, asking that a committee be appointed to act in conjunction with eight other gentlemen, to get up some suitable memorial for Dr. Atkinson. I think a resolution should be made to that effect, and I move that a committee of two be appointed for that purpose.

Seconded and carried.

*The President.*—The appointment of that committee will be made at a future meeting.

We will now proceed to the paper of the evening, which will be read by George W. Weld, M.D., D.D.S. I have the pleasure of introducing Dr. Weld.

(For Dr. Weld's paper, see page 501.)

*The President.*—The subject of Dr. Weld's valuable paper is before you for discussion, gentlemen.

We are so fortunate as to have Professor Elliott, of the College of Pharmacy, with us this evening. We should be very happy to hear from Professor Elliott.

*Professor A. H. Elliott.*—Mr. President and gentlemen, I must apologize for appearing before you this evening, since I am not a member of your Society; but in the society of science I believe we know no sections. Professionally, I am a chemist, and know very little of the physiological questions that Dr. Weld has so ably expounded; but, some three or four years ago, he interested me in this subject, from a chemist's point of view, and some of his interesting experiments with enamel came to my notice. A short time ago he told me he had been making some further experiments, and asked me if I would make a chemical analysis of the same.

I took a certain amount of enamel,—I will not bother you with figures,—which, in a three-per-cent. solution of acetic acid, was dissolved to the extent of about thirty per cent. A question arose in my mind as soon as I had made the analysis. I had some of the same enamel that I had analyzed three or four years ago, which contained about eighty-seven per cent. of phosphate of lime, and about eight or ten per cent. of carbonate of lime. The result of the experiment with acetic acid was this: I not only determined that a certain amount of this enamel was dissolved, but I went further: I analyzed the solution in order to see if the ratio of dissolved material was the same as the ratio of those materials in the original enamel; that is to say, whether eighty-seven per cent. of the dissolved material was phosphate of lime. I do not know whether I told Dr. Weld this or not; I may surprise him when I say that there was not the same amount of phosphate of lime in the solution as there was in the original enamel, proving to my mind that there was more carbonate of lime dissolved than phosphate of lime. I do not know the particular reason Dr. Weld has in connection with this, but it appears to me that the cementing substance is carbonate of lime, for here we have a fractional solution, the carbonate of lime going into solution more readily than phosphate of lime. If each dissolved as readily as the other, the ratio of phosphate of lime to carbonate of lime would be the same as it is in the original enamel; but I found that the phosphate of lime comprised about eighty-four per cent. of the solution, while the original enamel contained eighty-seven per cent. and a little over, showing that there was a tendency of the carbonate to dissolve more readily than the phosphate in the short time given, about half an hour.

One other point: I also finished, this morning, another experiment that Dr. Weld asked me to make. He asked me to take the enamel, and just wash it for a few moments with the three-per-cent. acid, and note the effect. Soaking the enamel for half an hour in the acetic acid dissolves, as I have said, about thirty to thirty-three per cent. By merely washing and drowning the acid at once, I found one-eighth of the enamel dissolved; but I have not yet determined whether the ratio in this case consists of an increased solution of carbonate over phosphate of lime, but I strongly suspect it is so.

With regard to this interesting question of the Vichy water, its effect may be due, as Dr. Weld suggested, to the mechanical separation of the dissolving material from the enamel by the gas, which is a very common result in batteries. If a plate of copper and one of zinc be immersed in sulphuric acid,—I am talking purely from a chemist's point of view,—we will find in a few moments that the surface of the copper, below the fluid, is all coated very closely with bubbles. If into this we place a galvanometer, we will find that gradually the needle will drop off until it gets to zero. The reason of it is that there is want of contact between the fluid and this particular metal.

That is the mechanical explanation of the want of contact, or the want of action of the chloride of iron, in connection with Vichy water.

Another explanation is the possible formation of a sesqui-carbonate of iron, or the carbonate of iron that corresponds to the perchloride of iron. If to a solution of perchloride of iron we add carbonate of ammonia, the fluid may finally contain ninety-eight per cent. of absolute oxide of iron, and one and one-half per cent. of hydrochloric acid.

With care the hydrochloric acid can be made to unite with the carbonate of ammonia, so as to leave a very small amount of acid. Now, we can go further, and dialyze that. Take a solution of iron —peroxide of iron—without any acid in the solution, and with the acetic acid we can carry it down so that every one hundred parts will contain ninety-four, about, of peroxide of iron, and six of acetic acid.

In adding Vichy to this acid solution, it may not only act upon the free acid over and above that necessary to form a perchloride of iron, but it may go still further, and unite with the hydrochloric acid, in combination with the perchloride of iron, forming a percarbonate of iron, and that is possibly the reason for its fluidity and the ease with which it remains in solution.

That is all I happen to think of which has a bearing on the subject, but if there are any questions, I shall be pleased to answer them if I can.

Incidentally, I would like to mention a little experience. Dr. Weld mentioned fluids for cleansing the teeth. As some of you gentlemen may know, I have a good many things brought to me to analyze, and about a year ago a very nice lady came in bringing a solution, apparently clear, which had a slight ethereal odor. She wished me to make an analysis, and give her a certificate that it was not injurious to health. I examined it, and found that it contained eighteen per cent. of concentrated hydrochloric acid. The lady said she was selling a great deal of it for a tooth-wash, but she wanted my certificate that it was not injurious to health. Of course, it was not injurious to health, but it was injurious to teeth. I asked her if she had used it on any teeth, and she said yes, she was using it herself. Her teeth did look very nice and white, but I considered it an outrage that such things should be foisted on the public, and, of course, I did not give her a certificate.

*Dr. C. E. Francis.*—A lady came into my office not long since, bringing a small vial containing a colorless fluid, and asked if I could tell her what it was. She said that parties were going about down-town selling vials of this kind, at the price of one dollar per bottle, or six bottles for five dollars. It held about half an ounce.

A friend of this lady, a banker, bought six vials for five dollars, and presented them to several of his friends, and she had received one from him. I removed the stopper, and at once observed the fumes and recognized the odor of hydrochloric acid. I then dissolved a little carbonate of soda in some water and added a few drops of the fluid, which immediately effervesced. I informed her of its nature and warned her not to use it. I would remark, Mr. President, that some of the most marked cases of decalcification of human teeth I have ever witnessed have been the result of the action of lactic acid. Within a few days, a gentleman called upon me whose teeth I have watched for a long time, and it is perfectly astonishing to see how they melt away, much like sticks of candy immersed in water. He has been living on a milk diet for a long time, and his teeth are almost gone. They are so melting away that nothing will save them.

A lady brought a little child into the office of Dr. Phillips, one of my associates, some time ago, and was anxious to know why the little girl's teeth decayed so rapidly. Dr. Phillips called me in to look at them, and I observed that the child's teeth were sadly eroded. I asked if her child was taking any solution of iron or eating much acid fruit; she said no; then I asked her if she gave the child milk to drink at night just before going to bed. She replied, "Why, yes. I give her a glass of milk every night." The erosion was undoubtedly caused by the action of lactic acid upon the teeth. In such cases I give caution and advice, hoping to avoid such results.

*Dr. E. A. Bogue.*—Is this the time at which people ask questions?

*The President.*—Yes, sir.

*Dr. Bogue.*—I have been exceedingly interested, since Dr. Weld read his first paper here a couple of years ago, not only in the subject now being discussed, but also in another which he touched upon just as he finished his paper to-night, but upon which I confess myself endarkened rather than enlightened. The point is this: We hear a great deal about the action of acids that are taken as remedies. We hear, also, in this instance, from Dr. Weld, of the action of the salts of iron. I failed to get from him what the indirect action was. He certainly said that the fluids of the body were not changed,—those which were alkaline were not made acid,—but I did not understand him whether the remedies themselves were not discharged through the salivary glands, and left in the mouth in such a state that free acid might be found.

Inorganic medicines, many of them, are decomposed in their passage through the system. If I might understand him more correctly I should be very much obliged.

*Dr. Weld.*—Mr. President, I thought I made myself clear. I only alluded to that point from a physiological point of view. As I said, chemical observation in the future may determine that the secretions from the salivary glands may be rendered acid enough—if that is what Dr. Bogue means—to affect the teeth, but I do not think that is possible. If it be possible, I should be very happy if Dr. Bogue would explain it from a physiological stand-point. If I understand what the doctor means, it is that the blood is changed from an alkali to an acid by the ingestion of acids (an impossibility), and the acidity of the fluids of the salivary glands thereby increased. I do not think that could be made possible by the ingestion of acids. The tears and perspiration are always acid in reaction, and I think all the alkalies in the city could not change their chemical reaction.

Urine is acid. It is changed because the kidneys eliminate the alkalies. It is changed from an acid to an alkali, but then, as I stated, if the alkalies are stopped, urine immediately returns to its normal acid condition; but it is a very different thing with the blood, which is always alkali in reaction.

That is the point, and I do not see how to go to work to experiment unless it be really by clinical and chemical observation, and that would require a great deal of time; and while I am not able to say, from chemical observation, that such a thing may not occur, at the same time I am inclined to think it an impossibility. If Dr. Bogue will try it on himself and report, I shall be very glad to know the result.

*Dr. Bogue.*—I did try it, and the result was that the acid was distinctly perceptible in the saliva. I should like to ask Dr. Weld about the experiment of Dr. Chase, of St. Louis. He examined some cows fed on slops, and found that the saliva, which ought in its normal condition to be alkaline, was acid. He took some other cows, fed in the field, shut them up, and found, upon feeding them slops, that their saliva became acid, but upon feeding them hay the saliva became alkaline and remained so during the act of mastication, but after the meal was finished the saliva soon returned to its acid condition. Dr. Weld has alluded to the alkalinity of blood, and also the acidity of the urine after being passed through its process of secretion. Then why should not human saliva change from alkaline to acid and back again? I believe it does. In my own case, although I tried it several times, I did not go through a series of experiments. I found the saliva acid after using iron for a time, and I see no reason why we should not expect it.

Again, I have noticed the condition of patients coming from the German iron springs; and the amount of dental troubles arising after a course of treatment from these springs is marvellous. Disintegration at the margins of the gums and of the fillings. In the cases of some persons of whom records have been kept for years anterior to their course of treatment, one summer has shown a manifest breakdown. Dr. Weld confesses that he is unable to explain. Perhaps Professor Elliott will help us out; it is a subject of great interest, and one which we are all anxious to hear about.

*Professor Elliott.*—The gentleman who has just sat down has called attention to the action of mineral waters upon the teeth. There are two classes of mineral waters in which iron may occur. The first of these is a water in which iron is present as a bicarbonate of iron, and in the other case the water contains sulphate of iron. I think I am right when I say the water of Oak Orchard, in this State, is an acid water, in which the iron is in solution in the form of both chloride and sulphate of iron, while the High Rock Spring, of Saratoga, some years ago, at least, contained a bicarbonate of iron.

Now, with regard to the water containing bicarbonate of iron, I think we are in much the same position that we are with the Vichy water and tincture muriate of iron. But with the other waters we have a corrosive agent that is not so easy to counteract. It might be that the water containing the perchloride of iron, as I think many of the German iron springs do, could be safely taken in conjunction with Vichy water, but by drinking the water alone I should think it would be very likely to affect the teeth. If it will dissolve iron, it will very likely dissolve the enamel. A water of that character put into a boiler in which hard water has been used will dissolve all the incrustation. With regard to the secondary action of these acids, a great deal depends upon which acid is in excess. If we take a solution of nitrate of potassium, mix it with acetic acid, and then throw into it metallic copper, the copper will dissolve and give off red fumes, showing that the acid is dissolving the copper to form nitrate of copper. If, on the other hand, the solution be diluted, there would be no nitrate of copper formed, so it is largely a question of concentration.

One other point that occurs to me in connection with the possible advantage of using Vichy water with a tincture of muriate of iron would be that, while it would protect the teeth from any corrosion, owing to the fact that it is there as a sesqui-bicarbonate only, as soon as it gets into the stomach, the hydrochloric acid being so much more acid, the iron returns at once to its original condition as it would be in the original tincture of muriate. So, while the Vichy water allows it to pass the teeth, as soon as it gets to the stomach it returns to its original condition.

*Dr. Bogue.*—Allow me to express my thanks to the gentleman, for he has exactly answered the question. Now another question: Is anything known by which we may so protect ourselves against this acid reaction, so that we may get the therapeutic effect on the system at large and yet not on the teeth? The direct action I confess to have looked upon almost with indifference. I have failed to see how my swallowing half a dozen oysters with some lemon-juice on them has done me any harm. On the contrary. Yet I should hesitate to expose my teeth to the action of a lemon while I was chewing. I should not hesitate in using muriate of iron if I needed it; but I should expect exactly what was told to us. After it was sent into the stomach and then sent on its journey through the circulation, I should expect that the mucus in the mouth would be intensified in its acidity.

*Professor Elliott.*—I did not mean to imply that the sesqui-carbonate of iron in passing into the stomach would necessarily cause a secretion of mucous acid in the saliva. I am not enough of a physiologist to argue that point, but I should think not. For we know that, although we may take such substances as cream of tartar, we will find, if we examine the urine, that it will be alkaline. These organic acids are decomposed from the physiological processes, and the alkalies are secreted by the urine, and pass out by the kidneys as an alkaline fluid.

In regard to the question of some method of using these and mineral waters, there is the one already suggested by Dr. Weld,—that is to say, if you have an acid water, mix it with Vichy water to an agreeable extent, or to an extent that shall counteract the direct acid action, and produce bicarbonate of iron. It is difficult to prove that it is so, because it is in solution, and it is in the same condition that bicarbonate of calcium is in the mineral waters. We know that we cannot dissolve carbonate of lime in water to any reasonable extent. We know that precipitated chalk stirred in a little carbonic water will give a perfect solution. I would suggest that an alkaline Apollinaris, Vichy, or any of these waters, could be used containing bicarbonate of sodium in connection.

*Dr. Geo. S. Allan.*—Dr. Weld very kindly brought me yesterday one of the teeth he had decalcified in a one-half-per-cent solution of hydrochloric acid, and asked me to examine it microscopically. I took great pleasure in doing so last evening. Taking a small piece of the broken-down enamel, crushing it on a glass slab, and mounting it in the usual way, I found that the prisms were most beautifully separated from one another, and I was able to get a better view of the separated prisms than in any specimen I have ever seen.

There was no question whatever that the cementing substance in the prisms had been dissolved out in preference to any action having taken place on the enamel. The prisms could be seen perfectly formed, with pointed ends and wavy outlines. They were more translucent in character than I expected to see them, but most beautifully brought out. Some of the substance of the prisms had been dissolved, but there is no question but what the action of the acid had been mainly expended in dissolving the interprismatic cementing substances. I do not think these specimens either uphold or militate against the reticulum theory. They have nothing to do with it. So small a portion of organic matter would very easily be lost sight of in a specimen of this kind. It was simply interesting, as showing how very beautifully this weak solution of acid dissolves the cementing substance, and liberates the prisms in their original condition for study.

*The President.*—I hope that this subject will be still further discussed. Dr. Meriam, of Salem, is with us and we would like to hear from him.

*Dr. Horatio C. Meriam.*—I think, Mr. President, that the subject must be finally considered in a wider range, not only as it affects saliva from the mouth, but the saliva of different conditions. The saliva during mastication and the mucus that is secreted at other times are altogether different. One has been subject to heat, and the other has been produced by muscular activity and has undergone no chemical change. We must also distinguish between the fluids produced in a healthy circulation of all fluids in the body and those produced during a sluggish condition.

I have questioned many of my patients regarding their condition, when I have found extreme decay at the margins of the gum, and in almost all cases I have found that they were costive. This condition has been connected with mucus so tenacious that it could be drawn from the lips a foot or more before the line broke. If the teeth are coated with a mucus so tenacious and of sufficient acid strength to act on the teeth, that brings us to other questions.

We have another form of erosion that is produced in rheumatism. Two patients I have in mind now, where the bicuspids and all the incisors look as if they had been cut and polished. All the fissures are obliterated. It is clearly the acid from the glands at the end of the tongue acting on these teeth.

I have never felt that the action of iron produced the harm that we thought, because it seemed to me impossible that it should lodge in the positions where we find those cavities in quantities sufficient to make them. "The fathers have eaten sour grapes and the children's teeth have been set on edge," not on the sides; and so I have felt that we should go back to the condition of the saliva, to that of the mucus glands of the mouth, and what produced their condition.

The use of tannin to arrest decay has been advocated. We know that decay cannot be arrested by it, but we believe that tannin could have an action on the glands of the mouth, so that the fluids secreted would be more healthy. In some cases where there has been an erosion of the teeth, showing a corrugated surface, I have stimulated the mouth with alcohol, directing my patients to get a burning sensation night and morning, for the purpose of reaching these glands.

There is another condition,—that coming from the growth of germs in the mouth. Botanists have, I know, advised farmers to sow certain plants for the sake of securing the action of their roots in breaking down the soil, and thus preparing it for a more valuable plant. The roots, as you know, exude an acid which acts on the otherwise refractory soil. It is interesting here to recall the experiment where a plant was planted in a glass cylinder which was placed on a slab of polished marble; after the roots were seen to have reached and covered the marble, the cylinder was removed, and the outlines made by the roots could be traced by the etching caused by their excreted acid.

This subject is an interesting one, and I thank you for calling upon me.

*Dr. Barrett.*—It seems to me that we are endeavoring to establish from a single fact, or from two or three simple truths, a whole system of philosophy. In many cases we confound cause with effect, misapprehending both. Within my own observation I have never seen anything that led me to entertain the slightest apprehension of evil from any of the mineral acids. We do not find them in sufficient strength, or in such conditions, as to dissolve or affect the teeth. It is the organic acids, which are formed in the mouth and presented to the tooth-tissue in a nascent condition, that work the mischief.

There is another important fact to be considered in this connection; a point that was raised by Dr. Bogue and partially answered by the chemist. When acids are taken into the system they are at once decomposed. If we absorb acids continually, all the fluids and secretions do not necessarily become acid. There is a disruption of the elements of the acid compound, and new combinations are formed under the dominion of physiological law, and these take the place of the former. Thus we may imagine the hydrogen, the oxygen, and the sulphur of sulphuric acid parting company and entering into new combinations which would be of a distinctly opposite nature, and thus an alkaline reaction be established. Indeed, I believe that one school of medicine bases at least a part of its practice upon the reactions of nature, and for an acid condition prescribes, not an alkaline, but an acid remedy, according to their law of "similia similibus curantur." I am not advocating homœopathy, but I believe that this one principle recognizes the fact, whether consciously or unconsciously, that nature works upon methods of its own, and that to give any inorganic material is to invite an activity which can have no result except to disrupt it, if a compound, and to select from it such elements as can be used in the forming of other compounds that may be assimilated. If the inorganic matter ingested be a simple substance, it usually is excreted in precisely the same form in which it was received.

The saliva is not, then, made acid by the drinking of acid drinks or the eating of acid foods, for those acids might be so decomposed in the body, so metamorphosed by the physiological action of the organs of the system, as completely to reverse that which we might naturally expect would be the case.

We do not readily comprehend that which takes place within the human organism, that peculiar process which we call nutrition, which works according to its own laws. The body does not take up inorganic matter for nutrient purposes, nor will it necessarily derive its acids from acid sources. The fluids are elaborated within the organism, and whether they be acid or alkaline depends upon that nutrient process, the assimilative condition, and not upon the character of the food ingested. In certain nutrient conditions the fluids of the body may have an acid reaction, even though the most scrupulous care be exercised to take none but alkaline foods and drinks, and possibly because of that selection. There is a metamorphosis within the body that is not governed by the nature of that which is received from without, but which obeys behests that are subjective rather than those that are objective in their character.

*Dr. S. E. Davenport.*—I feel that we are under great obligations to Dr. Weld for his interesting and scientific paper, also for the experiments which he has performed so successfully before us, and I would move a vote of thanks to him for his efforts; also to Professor Elliott for giving us the fruit of his study in this direction.

Seconded and carried.

Adjourned. S. E. DAVENPORT, D.D.S., M.D.S., *Editor New York Odontological Society.*

