# Errata

**Published:** 1891-09

**Authors:** G. W. Weld

**Affiliations:** New York


### Domestic Correspondence.

### ERRATA.

TO THE EDITOR:

DEAR SIR,—In the discussion of the paper on "The Injurious Effects of Vegetable and Mineral Acids," etc., before the Odontological Society (see page 552, in the August issue), I have read as follows: "The tears and perspiration are always *acid* in reaction, and I think all the *alkalies* in the city could not change their chemical reaction." What I intended to have said is just the reverse,—viz., "The tears and perspiration are always ALKALINE in reaction, and I think all the ACIDS in the city could not change their chemical reaction.

G. W. WELD.

NEW YORK, August 3, 1891.

